# Retraction: An efficient post-processing adaptive filtering technique to rectifying the flickering effects

**DOI:** 10.1371/journal.pone.0323914

**Published:** 2025-05-01

**Authors:** The PLOS One Editors

The *PLOS One* Editors retract this article [1,2] due to concerns about:

- Compliance with PLOS policies on Submission and Publication of Related Studies;
- Similarities to a previous publication [3];
- The use of terminology that differs from standards in the field (e.g. “ancient rarities” instead of “artifacts”);
- The reliability of the reported results and conclusions.

AG, SV, NZJ, AN, and MA did not agree with the retraction. JSS and YN either did not respond directly or could not be reached.

The retracted article [1] was removed from the *PLOS One* website at the time of retraction due to the similarities with [3]. The article’s Copyright and Data Availability statements were also updated at the time of retraction, and the removed contents are no longer offered under the Creative Commons CC0 public domain dedication.
